# Investigating public values in health care priority – Chileans´ preference for national health care

**DOI:** 10.1186/s12889-021-10455-y

**Published:** 2021-02-27

**Authors:** Alicia Núñez, Chunhuei Chi

**Affiliations:** 1 Department of Management Control and Information Systems, School of Economics and Business, Universidad de Chile, Diagonal Paraguay 257, Office 2004, Santiago, Chile; 2 College of Public Health and Human Sciences, Oregon State University, 013 Milam Hall, Corvallis, OR USA

**Keywords:** Public preferences in healthcare, Communities, Priority setting, Resource allocation, Chile

## Abstract

**Background:**

This study aims to assess preferences and values for priority setting in healthcare in Chile through an original and innovative survey method. Based on the answers from a previous survey that look into the barriers the Chilean population face, this study considers the preferences of the communities overcoming those barriers. As a result six programs were identified: (1) new infrastructure, (2) better healthcare coverage, (3) increasing physicians/specialists, (4) new informatics systems, (5) new awareness healthcare programs, and (6) improving availability of drugs.

**Methods:**

We applied an innovative survey method developed for this study to sample subjects to prioritize these programs by their opinion and by allocating resources. The survey also asked people’s preferences for a distributive justice principle for healthcare to guide priority setting of services in Chile. The survey was conducted with a sample of 1142 individuals.

**Results:**

More than half of the interviewees (56.4%) indicated a single program as their first priority, while 20.1% selected two of them as their first priority. To increase the number of doctors/specialists and improve patient-doctor communication was the program that obtained the highest priority. The second and third priorities correspond to improving and investing in infrastructure and expanding the coverage of healthcare insurances. Additionally, the results showed that equal access for equal healthcare is the principle selected by the majority to guide distributive justice for the Chilean health system.

**Conclusions:**

This study shows how a large population sample can participate in major decision making of national health policies, including making a choice of a distributive justice principle. Despite the complexity of the questions asked, this study demonstrated that with an innovative method and adequate guidance, average population is capable of engaging in expressing their preferences and values. Results of this study provide policy-makers useful community generated information for prioritizing policies to improve healthcare access.

**Supplementary Information:**

The online version contains supplementary material available at 10.1186/s12889-021-10455-y.

## Background

Governance has emerged as an important issue in a national health system in recent years [1–3]. In a democracy, citizen’s participation in key decision making is an important part of governance. Among the key decision making in health systems, priority setting is recognized as one of the greatest challenges faced by health policymakers globally [4, 5].

Mechanisms for allocating limited resources can be classified into two major approaches, which are, implicit versus explicit mechanisms. An implicit mechanism is to rely on the free market serving as the “*invisible hand*” to allocate resources to those whose willingness to pay commensurate with the social valuation, i.e., the market clearing price, of such resources. The underlying assumption of market mechanism is that competitive market clearing price will serve as the “*invisible*” priority setting mechanism, and there is no need for external intervention.

In most nations with a state operated universal health system (such as National Health Insurance or National Health Services), it precludes the implicit allocation of health services by market. Yet, in most countries, universal care does not exist and/or the financial resources are low, and therefore, the limitation of resources leads to implicit rationing through waiting lines, low quality, inequities, and other mechanisms [6]. Most rationing is done implicitly at the patient level when the staff may not be aware that their decisions are rational decisions [7], taking place at the clinical or micro level [8]. The alternative to implicit mechanism of resource allocation is an explicit mechanism. An explicit mechanism of resource allocation for health care relies on the state or an institution that represent the public to allocate health care services for the people. Because of resource constraint, such explicit mechanism, inevitably, will have to determine what health care services will be provided to whom.

Inherent in such explicit allocation mechanism is the following assumptions:

- *Assumption 1*: Resource constraint in a health care system makes it impossible to provide every type of known health care service to everyone.
- *Assumption 2*: State intervention in resource allocation is desirable.
- *Assumption 3*: Not all health care services merit equal claim for resources.

From the above discussions, it is clear that the real question in health care resource allocation is not *whether*, but *how*, to set priority. On the *how* question, the prioritized list for health services under the Oregon Health Plan was the first attempt to set an explicit priority for health care services by the state [9]. Its underlying methodology has generated great interest around the world ever since [10, 11]. Most notably, from Oregon’s experience in the late 1980s, both researchers and policy makers gradually recognize that the nature of health care priority setting is normative, and therefore is largely a political process, instead of a pure technical operation. With this understanding, researchers have developed various methods to address the need of soliciting public values in health care priorities.

In a democracy, these political dimensions require engaging community-level decisions around shared values and preferences [5, 12–14]. Engaging citizens directly in these decision-making processes strengthens legitimacy, transparency, and accountability, which in turn increases the public’s sense of ownership in the policy and trust in the related outcomes [15, 16]. Since the late 1980s, many governments have recognized and begun to operationalize the notion that priority setting should incorporate, or primarily rely on community preferences that can be analyzed alongside technical data [17]. There remains, however, the question of how to implement credible methods to capture this perspective.

The general approach of ‘stated preference’ models, through which the consumer can explicitly compare, weigh, and state preferences between health services [18], serves as a starting point for developing various methods to collect public preferences. Capturing societal values however offers distinct challenges in defining and implementing as they involve subjective judgments and can be interpreted differently across individuals and communities. Research into effective methods to solicit, measure, and aggregate community values is increasing, yet is still underdeveloped, which motivated this research to examine such methodology.

Researchers have developed various methods for soliciting public preferences or values over the last two decades. Most of the methods were developed by health economists, with a few by other social scientists and health service researchers. Health economists in general favoring soliciting quantitative information or data through sample survey that allow them to perform sophisticated statistical analysis, while other social scientists also favor qualitative methods. Of the survey-based quantitative methods, followings are the commonly used methods:

1. Contingent Valuation (CV),
2. Choice Modeling,
3. Conjoint Analysis,
4. Discrete Choice Experiment (DCE), and
5. Multi-Criteria Decision Analysis (MCDA).

Among these methods, DCE has been the one that seems to be most popular among health economists [19–22].

For qualitative methods used to solicit public preferences, followings are the commonly used ones that based on various deliberative process:

1. Citizens’ Jury (CJ),
2. Deliberative Conferences,
3. Deliberative Mapping,
4. Deliberative Polling, and
5. Deliberative Workshop.

Among these methods, CJ has been one of the most popular methods for soliciting public values or preferences in health care [23, 24].

While many researchers continue to improve these two groups of methods to address various limitations, ranging from community representation, reliability, validity, to reconciling philosophical and epistemological inconsistencies, there is one additional dimension of the challenge. That is, a simpler method to administer and understand by the general public with a minimal level of education (such as elementary/primary school level), which does not require extensive time or multiple encounters to conduct, and can accommodate a larger sample. At present, most of the popular methods in both quantitative methods (such as DCE) and qualitative method (such as CJ) do not meet this criteria. The motivation for this research, therefore, is to explore an alternative method for soliciting public values and preferences in health care that meet this criteria. This experiment is a new contribution to the discussion of involving general public to participate in priority setting [25–29].

For this purpose, the continuous evolving and innovative Chilean health care system is the appropriate context to explore this new method. The Chilean health care system shifted in 1981 from a mainly public funded system to a mixed system of public and private insurance, which effects include creating a gap between income groups (high income groups migrated to private insurance companies), and reductions in government subsidies, causing the deterioration in the provision of services. With the re-democratization of Chilean political system in the 1990s, it also opened up new ideas and provided opportunities for Chilean health care system to explore and reform. Thus, one major health care reform was introduced in the 2000, creating a plan called AUGE (Regime of Explicit Health Guarantees). AUGE (now GES) is a health program that benefits all Chileans without discrimination of age, gender, economic status, health status, or place of residence. Along these reforms, there is also an increasing sense of need to improve the system and the governance of the Chilean health system, including promoting public participation in key decision making [30, 31].

It is in this background that we decided to conduct this exploratory investigation in the context of the Chilean society and its health care system. That is, in this research we seek to explore an innovative survey method that incorporates some key features of trade-offs considerations for survey subjects to decide their values and preferences in health care priority for Chile. An important purpose of this study is to experiment and assess whether the average people in Chile (and, for that matter, in most countries) is capable of making choices of health program priority, and also expressing her/his preference for a principle of distributive justice to guide priority setting.

## Methods

### Methodology rationale

In the last section we discussed that the nature of national health care priority setting is primarily in the normative domain. As such, in a democracy, a natural approach is to seek public input and participation. In this section we will discuss some philosophical foundation for our research.

The two common approaches in soliciting public preferences, quantitative survey-based methods, and qualitative-deliberative methods, each has its own ethical and philosophical foundation and assumptions. For quantitative survey-based methods, it is primarily built upon the assumption of philosophical individualism. That is, researchers and policy makers consider that a society is merely an aggregate of individuals, and there is no such notion as “collective self of a society” [32]. Most researchers who employ this method tend to implicitly make this assumption. Further, survey sample subjects who participate contribute with their ideas or preferences as *individual qua individual*, with little or no consideration of community. Likewise, the quantitative analysis that is applied to analyze the data is also largely methodological individualism. In order to obtain the preferences of a society or community, one simply aggregates the preferences obtained from individual samples. Technically, it is feasible to organize survey-based method based on other philosophical foundations about society other than individualism, such as communitarianism. To do so, researchers will need to include extra steps in organizing the survey, such as informing the sample subject before the survey that they are being asked to consider their preference for the society, instead of themselves. Or, taking a step further, to provide survey samples to meet and discuss the survey questions before they take the survey. In addition, when analyzing the survey data, one also need to take extra steps to assigning community weights or other various methods of weighting, rather than a simple aggregation and let the majority voice be the community’s voice. In practice, this is seldom done.

On the other hand, qualitative-deliberative methods are frequently based on a communitarian notion of society and community. That is, a society is more than just the aggregation of individuals. In addition, a society is also an organic entity with a “collective self of a society” that influences the individual’s values and behavior; at the same time individuals also shape this organic social self. A major philosophical difference between individualism and communitarianism in soliciting social preferences is that in individualism sample respondents are providing their preferences as *individual-qua-individual*, while in communitarianism they are providing their preferences as *individual-qua-society* [33].

Whether a researcher chooses to base her/his research on individualism or communitarianism, the researcher is also faced with methodological challenges in choosing survey-based quantitative method vs. deliberative qualitative method. Both methods require extensive time commitment by the respondents that preclude a large sample study. DCE usually accommodate a sample size of a few hundreds, unless the research project has a large funding with big team, while CJ is limited to between 20 to 30 participants due to its extremely time consuming deliberative process and requirement for reaching a consensus or near consensus.

The DCE was originally developed using a specially designed survey to assess public preferences in the field of transportation in the 1970s. It has since become a leading method for soliciting preferences in numerous areas of study, especially health care priority setting [18–21] Respondents are asked to consider choice sets with numerous options and select the one that they prefer. These options are defined and differentiated in the questionnaire by varying levels of select attributes. In the choice sets researchers can include a ‘none of these’ or ‘status quo’ option. The researcher that performs statistical analysis on respondents’ varying preferences related to differences in attribute levels, and assess the relative strength of aggregated preferences and respondents’ willingness to make trade-offs across the selected attributes.

DCE allows quantitative estimation of the marginal utilities associated with a set of attributes, in contrast to other methods that simply aggregate measures such as burden of diseases. These estimations provide additional information on relative preferences around these attributes that is helpful for decisions around resource allocation priorities. This method has been applied to a wide variety of questions in health policy, from public preferences around prevention versus treatment to policymaker trade-offs between equity and efficiency [34, 35].

There are at least two related limitations of implementing DCE in a larger population. The first one is the structure of survey in DCE is by nature complex, and therefore, time consuming to administer. Another limitation is the survey generates many data points per sample, together with the time consuming nature, makes it resource (funding and time) intense if a researcher would like to administer it to a larger (more than a few hundreds) sample.

For this reason, this research explores an alternative method we developed that is less complicated than DCE, easier to administer and understand by subjects with minimal education, hence can accommodate a larger sample. What we are proposing is not to replace DCE because there are many contexts in which DCE can serve the research purpose well. Rather, we are exploring an alternative method to solicit community preferences when research contexts require a larger sample, lower resources, and shorter time to complete.

### Research method

Based on the rationale discussed in the last section, we developed this alternative method of soliciting community preferences in health care, and experimented it in Chile. This research was part of a comprehensive research on the access to health care and Chilean people’s perception in overcoming access barriers of the Chilean health care system. We conducted a first survey were we identified barriers of access, then in a second survey (focus of our study) we collected information regarding the communities’ preference to overcome those barriers previously identified. The first survey was structured based on an ontology that highlights the many pathways for improving access within the health care system [36]. Barriers of access may come from different constructs such as the characteristics of the health care services, the utilization of services, the satisfaction with the services, the characteristics of the population at risk, and the health care policies [37]. In this way, every community was able to determine its specific barriers that set the basis to evaluate the relative inaccessibility to the system. This first survey included seven modules asking about demographic/geographic information and questions from all the combinations described in the ontology. The second survey developed for this study considers communities willingness to overcome the barriers detected, considering people’s values and preferences for priority, not only thinking about themselves as individuals, but also as members of the society and, therefore, considering the entire Chilean population and society, its application followed five steps: (1) selecting a representative subsample for three regions of Chile, (2) designing the survey, which involves focus groups for item development, formulation and reformulation of questions, and piloting testing, (3) administering the survey, and (4) analyzing the data.

#### Step 1: sample selection

Based on a sample defined from a first survey that identified barriers of health care access, a second survey was developed to establish priorities to overcome those barriers. Therefore, this is a panel-type survey with two waves that was designed and applied in three regions of the country (II, VIII and XIII). The three regions were selected using a convenience sample given their relatively large number of ethnicities, population size, and a mix of urban and rural areas.

The original sample included a total of 1885 interviews segmented on a total of 42 rural and 231 urban areas. We applied a multistage stratified random sampling with an application of the systematic simple random method in the selection of the units to be surveyed. The National Census was used as the sampling framework.

The stratification of the sample from the National Census allowed us better estimates and similar statistical errors in each stratum for comparison purposes. We also used clusters to improve the quality of the data collected to facilitate the identification of households and to reduce the time and cost of interviewers’ travel. The systematic simple random sampling allowed each household to have the same probability of being selected, and therefore a better dispersion of the sample. Finally, the selection method was multi-stage due to the existence of more than two sampling levels. Our last sampling unit was the individual who was randomly selected using a Kish Grid. A Kish Grid assigns numbers to each member of the household, based on age, to find the person to be interviewed. This grid assigns an equal probability of selection for each possible survey participant.

In this second wave we were able to reach 60.5% of the participants from the first wave. Therefore, this second survey includes a total sample of 1142 individuals, as displayed in Table 1.

**Table 1:** Survey sample

Region	Total number of effective interviews
II - Antofagasta	360
VIII - Bío-Bío	382
XIII - Metropolitan	400
Total general	1142

#### Step 2: survey design and piloting

The survey contains three sections. A first section includes six programs that will request the Chilean health care system to overcome the access barriers that were identified by the study sample on the first survey. These programs can be summarize as: (1) investing in new infrastructure, (2) providing better and more healthcare coverage, (3) increasing physicians/specialists, (4) adding new informatics systems, (5) promoting new awareness of healthcare and public health programs, and (6) improving availability of drugs. Sample respondents were asked to prioritize each of these programs; firstly by their opinion and secondly by allocating resources. The second section asks people’s preferences for a distributive justice principle for healthcare to guide priority setting of health care services in Chile. The principles included are: 1) equal access for healthcare, 2) equal access for equal health needs, 3) equal access for equal ability to benefit, and 4) equality in health. The final question is a public opinion question about respondents’ preferences in participating in major health policy decision making. All questions were piloted in a small-scale sample to revise the instrument. See in the supplementary material the survey instruments we used.

#### Step 3: survey administration

The survey was conducted during the months of August, September and October of 2018. Field work required a general project coordinator overseeing 3 regional field coordinators and 28 trained interviewers.

#### Step 4: data analysis

The data was analyzed using STATA version 13 and R software. We conducted frequency analysis and decision trees for assessing respondents’ preferences for prioritization.

### Survey instrument of trade-off

In order to incorporate the feature of trade-offs in priority setting (i.e., when a resource is allocated to program A, it is no longer available for any other programs), we develop a unique yet relatively simple instrument for our survey purpose. That is, we gave every respondent 18 identical stickers, with each sticker representing equal quantity of resources (unspecified funding). The respondents’ task was to allocate these stickers to those 6 programs; the more important they think the program is, they put more stickers on the program. At the same time, respondents know very clearly that there is only a limited number of stickers they can put on the program(s) of their choice. If they think all programs are equally important, they can distribute those stickers equally among these programs. In this way, respondents are setting priority with a budget constraint.

In our pilot testing, we experimented with providing respondents with hypothetical money as budget. Assigning priority with hypothetical money, however, created several complications that threatened the validity of our research. One of the side effect was respondents were thinking about real money, and for the program of their choice they feel it needs all the money it can get, and were more willing to put all budget in one program. Using hypothetical money, therefore, prevented respondents from considering all 6 programs in a more comprehensive way because they were preoccupied with the notion of whether their favorite program will get enough money. Another complication was some respondents, while thinking on real money term, considered some program does not need more money. These side effects can defeat our purpose, which is not to ask public preferences about exact budget allocation. Instead, we were focusing on investigating what were the public’s preference in prioritizing these 6 programs.

## Data and results

### Data

A total of 1142 completed surveys were collected from direct interviews with one selected individual per household. Up to three visits were made to each household in order to find the person to be surveyed. Among the total sample 650 (57%) were male, 491 (43%) were female, and 1 (0.09%) was a transgender. In our sample, 398 (35%) participants lived in the XIII Region, 358 (31%) in the II Region, and 386 (34%) in the VIII Region. In terms of working status, 337 (30%) of them were working for a salary, 282 (25%) were working in household tasks, 215 (19%) were retired or pensioned, 160 (14%) were students, 56 (5%) were looking for a job, 25 (2%) were disabled, 17 (1%) were volunteer, and 50 (4%) were classified as in any other working situation. As for respondents’ religions, 653 (57%) participants are Catholics, 245 (22%) are Christians, 127 (11%) are Agnostic, 59 (5%) Atheists, and 58 (5%) belonged to other religion.

In addition, most of our sample respondents did not belong to a specific ethnic group (1040 (91%)), while 50 (4%) were Mapuches, 36 (3%) declared to belong to a different ethnic group, 8 (1%) were Diaguita, and 8 (1%) were Aymara. In terms of education, the majority of participants have some level of education (1003 (88%) participants), while 139 (12%) participants have no formal education. Finally, in terms of health insurance coverage, 929 (82%) participants are subscribed to the public insurance (FONASA), 105 (9%) belonged to the private insurance (ISAPREs), 13 (1%) to the army insurance, 26 (2%) to other insurance, and 69 (6%) had none insurance.

### Results

A total of 1142 sample respondents took the survey to prioritize 6 programs (A to F) that will improve their access to health care. The participants can assign to more than one program the same priority but each program could only be assigned once. These programs included:

- **Program A:** Investment in new health care facilities to provide easier access (closer distance to places where survey subjects live) and reduce travel time.
- **Program B:** More generous insurance coverage of benefits (FONASA, ISAPREs, etc.) such as for prescription drugs, lab exams, alternative medicine and medical attention.
- **Program C:** Increase in the number of physicians and specialists available and improve their communication with patients.
- **Program D:** Investment in information systems that make reservation for appointment easier and faster.
- **Program E:** Improve the distribution of health care and public health awareness programs (e.g. oral health, sexual and reproductive health, mental health, etc.) to all regions and better dissemination of those programs.
- **Program F:** Improve availability of prescription drugs in all health care facilities and pharmacies.

The estimated total number of assigned priorities was 6852 (1142 respondents × 6 programs to be assigned); however; in one case, only 4 out of the 6 programs were prioritized, giving a total of 6850 responses. Table 2 presents a summary of priorities assigned by respondents. All 1142 respondents’ assigned one or more programs as their first priority, achieving 2304 answers that can be decomposed as 380 votes for program A, 361 for program B, 800 for program C, 208 for program D, 272 for E, and 283 for program F. Then, on the second step, 1025 respondents assigned one or more programs as their second priority, with a total of 1346 programs being assigned to this priority. Finally, the sixth priority, where 596 respondents assigned their priorities reaching a total of 596 responses.

**Table 2:** Summary of priorities assigned by respondents

Priority	Number and percentage of respondents						
	With priority assigned		Without priority assigned		Total		Total
	N°	%	N°	%	N°	%	N°
**1st Priority**	1142	100.0%	0	0.0%	1142	100%	2304
**2nd Priority**	1025	89.8%	117	10.2%	1142	100%	1346
**3rd Priority**	935	81.9%	207	18.1%	1142	100%	1028
**4th Priority**	829	72.6%	313	27.4%	1142	100%	852
**5th Priority**	719	63.0%	423	37.0%	1142	100%	724
**6th Priority**	596	52.2%	546	47.8%	1142	100%	596
**Total**							6850

We summarized the frequency and percentage of respondents who choose one or more programs as first priority. According to Table 3, more than half of the interviewees (56.4%) chose a single program as their first priority, 20.1% selected two programs as their first priority, while 10.2% pointed out that all 6 programs were equally important, and therefore choose them as first priority.

**Table 3:** Count of programs in first priority

Number of programs in First Priority	Frequency	Percentage	Cumulative percentage
**1**	644	56.4%	56.4%
**2**	229	20.1%	76.4%
**3**	117	10.2%	86.7%
**4**	26	2.3%	89.0%
**5**	9	0.8%	89.8%
**6**	117	10.2%	100.0%
**Total**	1142	100.0%	

Likewise, in the second priority, 69.6% of the interviewees indicated a single program as their second priority.

Table 4 shows the frequencies obtained for each priority. Results indicate that program C to increase the provision of physicians and specialists in the country and improving their communication with patients was the one that most respondents (34.7% of them) chose as their first priority. This is followed by Programs A and B with 16.5 and 15.7% respectively.

**Table 4:** Frequency analysis

Program	1st Priority		2nd Priority		3rd Priority		4th Priority		5th Priority		6th Priority	
	N°	%	N°	%	N°	%	N°	%	N°	%	N°	%
**A) Infrastructure**	380	16.5%	195	14.5%	147	14.3%	136	16.0%	132	18.2%	152	25.5%
**B) Better health care coverage**	361	15.7%	234	17.4%	182	17.7%	162	19.0%	142	19.6%	60	10.1%
**C) Physicians and specialists**	800	**34.7%**	216	16.0%	65	6.3%	29	3.4%	13	1.8%	19	3.2%
**D) Informatics Systems**	208	9.0%	180	13.4%	187	18.2%	178	20.9%	173	23.9%	215	36.1%
**E) Awareness health care programs**	272	11.8%	257	19.1%	245	23.8%	179	21.0%	134	18.5%	55	9.2%
**F) Prescribed drugs**	283	12.3%	264	19.6%	202	19.6%	168	19.7%	130	18.0%	95	15.9%
**Total**	2304	100%	1346	100%	1028	100%	852	100%	724	100%	596	100%

Subsequently, in order to investigate more about the quantitative differences between priorities to improve the population’s access to health services in Chile, respondents were asked to assign a score (stickers) to each program. Each respondent was given 18 stickers equivalent to 18 equal points, and was reminded that their choices of allocating these stickers will have an impact on access to health services. Our interviewers instructed them that to have an impact on health care services a respondent should assign at least 3 points or 3 stickers to a program (the 3 stickers cut-off point come from the 18 stickers available divided by 6 programs). However, the more points respondents allocate to a program, the better improvement Chilean people will get from that program. The results of this exercise are summarized in Table 5. The results indicate that the most valued program is program C, which received 30.5% of the total score that was doubling the score from other programs. In fact, Program C obtained 5069 points out of a total of 6278 from those who chose this program as their first priority.

**Table 5:** Priority scores by program

	Program A	Program B	Program C	Program D	Program E	Program F	Total
**1nd Priority**	1862	1563	5069	683	1048	1150	**11,375**
**2nd Priority**	525	661	839	404	698	778	**3905**
**3rd Priority**	313	404	202	317	557	518	**2311**
**4th Priority**	217	293	55	246	303	315	**1429**
**5th Priority**	168	191	22	176	160	172	**889**
**6th Priority**	116	37	19	170	70	104	**516**
**Total**	**3201**	**3176**	**6278**	**2026**	**2836**	**3037**	**20,554**
**Percentage**	**15.6%**	**15.5%**	**30.5%**	**9.9%**	**13.8%**	**14.8%**	**100%**

We also generated a decision tree to predict future preferences based on the conditions or characteristics of the individuals in this study. This method allows us to extract patterns to predict whether a “new” individual who has similar characteristics to those individuals in the sample will choose a specific program as a priority.

To execute the method, we created a dummy variable with 2 categories: “Yes”, for those individuals who want to see a greater impact in the program and assigned 4 or more stickers to it, and “No”, otherwise. The variables used for the program’s decision tree were: region, age, income, education, body weight, health insurance, ethnicity, nationality, chronic disease, healthcare utilization and gender. They were selected according to their relevance in the ensemble. With this method, patterns were extracted, which allow us to predict whether a “new” individual with the same conditions will choose Program “x” as a priority. We use the following equation in R:

$$ \mathrm{rpart}\left(\mathrm{C}\sim \mathrm{region}+\mathrm{age}+\mathrm{income}+\mathrm{education}+\mathrm{body}\ \mathrm{weight}+\mathrm{health}\ \mathrm{insurance}+\mathrm{ethnicity}+\mathrm{nationality}+\mathrm{chronic}\ \mathrm{disease}+\mathrm{health}\mathrm{care}\ \mathrm{utilization}+\mathrm{gender},\mathrm{data}=\mathrm{datos},\mathrm{method}="\mathrm{class}"\right) $$

**Fig. 1:**
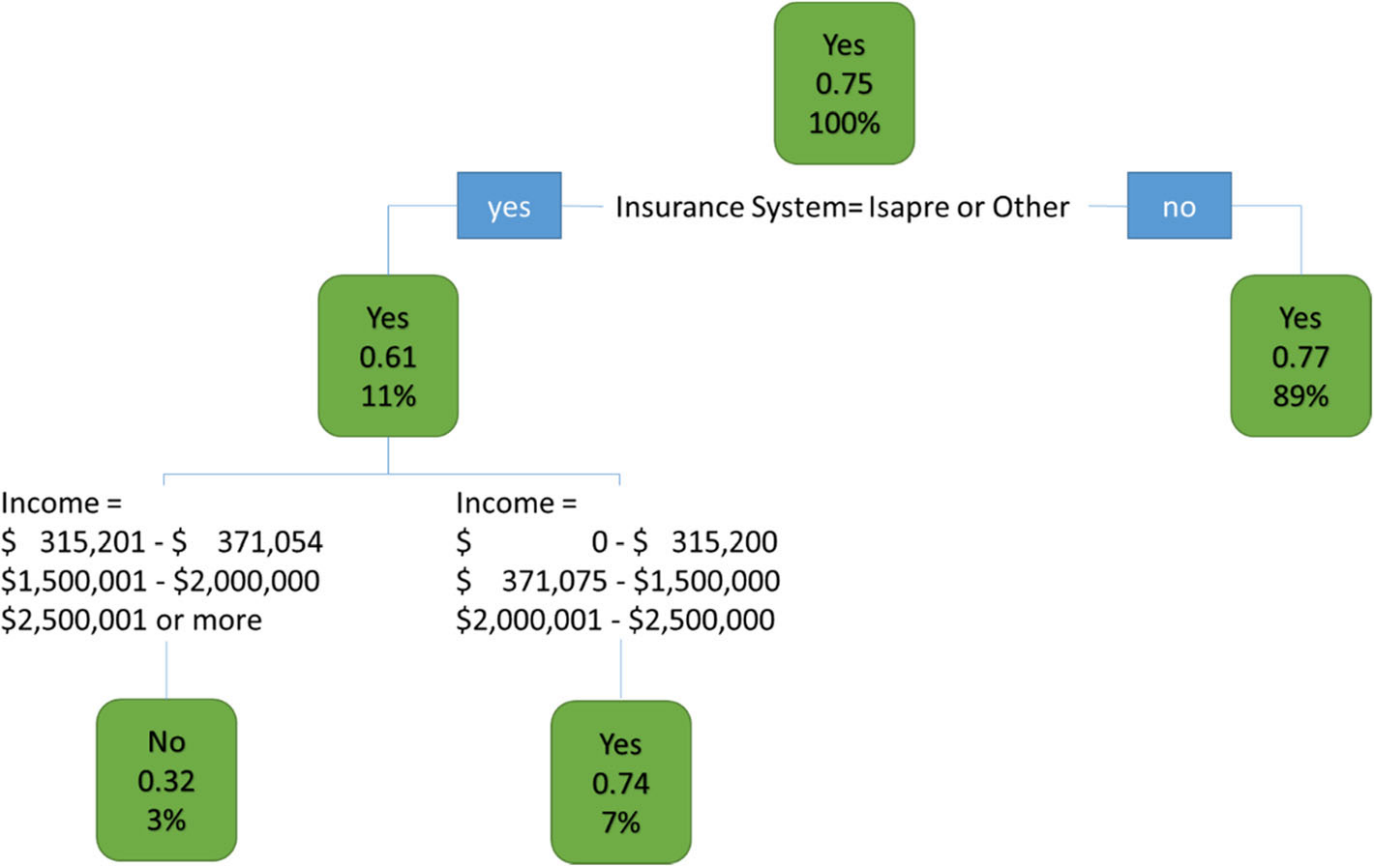
Program C pruned decision tree

Then, we prune the tree for better predictions and create a generalized model. Below in Fig. 1 we show the results for program C pruned decision tree.

Program C’s pruned decision tree considers two subgroups, 765 individuals in the working group and 377 individuals in the training group, adding a total of 1142 individuals. According to this tree, 75% of individuals (577 respondents) indicated Program C as their priority program. Of the 765 respondents, 82 respondents had a private insurance or other type of insurance. Among them 61% (50 people) chose Program C as their priority and 39% of them (32 people) did not. Further, among the 82 respondents who has a private insurance or other type of insurance, 25 of them have an income in the following categories: $315,201–$371,054 CLP, between $1,500,001–$2,000,000 CLP, or higher than $2,500,001 CLP. Among them 68% (17 people) chose Program C as their priority while 32% (8 people) did not. Additionally, 82 participants have an income in the following ranges: between $0 - $315,200 CLP, $371,075 - $1,500,000 and $2,000,001 - $2,500,000 CLP. Among them, 74% (42 people) chose program C as a priority, while 26% (15 people) did not. On the other branch of the tree, out of the 765 respondents, 683 have a public insurance, an army insurance or non-insurance. Among them 77% (527 people) chose Program C as a priority and 23% (156 people) did not.

Then, the second section of the survey asks about people’s preferences for a distributive justice principle for healthcare to guide priority setting of health care services in Chile. This is a broader question, not just defining the preferences for a particular program but looking at what communities perceive as fair on how healthcare should be distributed in the country. It is important to emphasize that the aim was not necessarily to provide the right answer but rather to assist in determining what ought to be the prefer distributive principle for the communities. Further, one of our purpose in this research was to assess whether it is feasible for the average people to understand the complexities of different distributive justice principles, and make a choice of them. We designed an innovative survey method that transformed the complex distributive justice principles into concrete and easily understandable example of policy implications following certain distributive justice principle. Our result is very promising, which indicate that the average people is capable of grasping these complex principles and make a choice. The principles included were: 1) *equal access for healthcare*, 2) *equal access for equal health needs*, 3) *equal access for equal ability to benefit*, and 4) *equality in health*. We understand that there are more than those four principles of distributive justice for equity in health care. Upon reviewing the literature, we choose these four principles based on both the popularity of the principle and relatively easy to understand by the average people, and also avoid confusion for some principles that are closely related to one of these four principles [38–41].

We foresee that this is an abstract ethical question that not every sample respondents can easily understand, let along making a choice. In addition to a clear definition of what each distributive justice principle is, therefore, we also designed an innovative method that includes:

1. Pictures of 3 persons with different age, gender, and health conditions (Table 6)
2. A Question card showing the implications of choosing each distributive justice principle and how it affects these three representative persons in an example of heart transplant with only one available donated heart (see Table 7).

**Table 6:** Sample representative persons for distributive justice question

Person	Age	Gender	Heatlh Conditions
A	35	Female	Heart failure, needs a heart transplant
B	75	Male	1.High blood pressure 2.High blood cholesterol level 3.Alzheimer’s disease 4.Heart failure, need a heart transplant
C	20	Female	None (healthy)

**Table 7:** Descriptions of implication of choosing each distributive justice principle

Options	Distributive Justice Principles	Persons who will receive the heart		
		A	B	C
1	Equal access for health care	✓	✓	✓
2	Equal access for equal health needs	✓	✓	
3	Equal access for equal ability to benefit from health care	✓		
4	Equality in health	✓^a^		

^a^ Depends on how we measure health

With this method, all respondents were able to make a choice, otherwise, it will be a very abstract question. These pictures of representative persons, and an example of resource allocation (donated heart for heart transplant) help our respondents to see the concrete consequences of choosing a distributive justice principle. Results indicate that there is no clear majority preference to establish which should be the distributive justice principle guiding healthcare resource allocation in the country. The principle that received the highest percentage (33%) was *equal access for healthcare*, which is closely followed by the principle of *equal access for equal ability to benefit* (29.1%). We summarized the results in Table 8.

**Table 8:** Distributive Justice Principle

Answer	Frequency	Percentage
**Equal access for healthcare**	378	33.1%
**Equal access for equal health needs**	264	23.1%
**Equal access for equal ability to benefit**	332	29.1%
**Equality in health**	168	14.7%
**Total**	**1.142**	**100%**

Finally, we included a public opinion question regarding whether the Ministry of Health should ask the Chilean people about their opinion in major healthcare system policies. The answer was an overwhelming majority (95.4% of respondents) would like to be asked about their opinion, while 1.8% answered they would not like to participate, 0.5% provided an indifferent answer, and 2.2% of the participants did not know.

## Discussion

In this research we investigated Chilean people’s preferences in the priority of improving access to health care, based on 6 priority programs identified in our previous study. By applying an innovative survey method, we demonstrated that this method can be implemented to a large sample that included respondents with minimal level of education. Further, the use of points allowed respondents to make conscious trade off in choosing their priority program(s).

The method we explored is still largely survey-based quantitative method, without the complicated and time consuming features of other methods like DCE. It lacks the deliberative process that is critical to CJ. What we attempted was to incorporate a sense of communitarianism into the survey method that is largely methodological individualistic, by asking our survey subjects when they are considering values and preferences for priority, not to think only about themselves, but also to think about the entire Chilean population and society. That is, what Mooney [33] called “*individual qua society*” instead of “individual qua individual”.

Results of our simple question survey was validated with our trade-off points` allocation survey. In both cases, Program C that will increase the number of physicians and specialists available and improve their communication with patients got the first priority by our sample respondents. Further, our experiment suggests that with a simplified survey method, average community members are capable of making complex choices. Likewise, our use of pictures to represent persons of different age, gender and health needs, together with a concrete example of allocating a donated heart for heart transplant was successful in soliciting every respondents’ answer of this difficult choice. It suggests that with appropriate design and a concrete example, average public is capable of making choice of an abstract nature, such as distributive justice principles.

These findings are in line with the deficiencies identified in the country where the public sector shows lack of resources and specialist physicians in the metropolitan area (the capitol) but particularly in the rural regions of the country [42]. It is important to notice, that we are not considering the actual costs of implementing the health program or programs, and should be a matter of study in the future. Instead, we are assessing community’s preferences within a budget constraint.

Our results on preferences for a distributive justice principle to allocate health care resources, on the other hand, was not conclusive. We suspect that the concept of distributive justice for allocating health care resources might be too complicated for a survey, despite that we used figures and hypothetical choice consequences in the survey. Also, a limitation of this study is excluding some important principles of distributive justice in health care, such as health maximization that is based in utilitarianism, and forms the rationale for allocation of health care resources based on cost-effectiveness analysis and value for money. However, there are rooms for improving survey method of soliciting community values on more complex and somewhat abstract concepts such as distributive justice principles. Further, for a question like choosing a distributive justice principle, it might be helpful to supplement our survey with a CJ. A CJ result will help validate or modify the findings from survey.

Results of this research also suggest that Chilean people is capable, and would like to participate in key decision making of Chilean health care system. For example, when considering priority setting in health care, the Ministry of Health may want to implement a method for Chilean people to participate and have their voices be heard. That method could be DCE, CJ, or a simpler survey such as the one we introduced.

### Study considerations

This study includes three out of sixteen regions from Chile. However, they correspond to regions that have a high concentration of population, close to 51% of the country’s total population. Our subsample did have demographic characteristics similar to those of the original population.

### Policy implications

It is essential for policy makers to understand both the barriers faced by the population and the priorities placed by the same population to overcome those barriers when trying to assess and improve the healthcare system. Today the Chilean healthcare system does not guarantee receipt of all necessary care, and therefore, there’s call for changes. Indeed, request for improving equity in healthcare was one of the focus of mass protests in Chile that took place in October of 2019 [43, 44]. Also, according to our results, there is sample room for improving physicians’ communication with patients. By improving physicians’ communication with patients, it can prevent subsequent visits or leave patients with doubts about their treatments. Policy reforms need to address all these issues mentioned in the 6 programs, while our results shows what Chilean people’s priorities are.

## Conclusion

This study proposes an alternative method using a survey for soliciting public values and preferences in health care, allowing the participation of a large sample of individuals.

Despite the complexity of the questions asked, this study demonstrated that with guidance, population can express their preferences and values, providing policymakers with valuable community generated information for decision-making, and expanding the debate on health to establish policies that help to have a more equitable system and a better community perception.

## Supplementary Information

**Additional file 1.** Survey.

**Additional file 2.**

## Data Availability

The datasets used and/or analyzed during the current study are available from the corresponding author on reasonable request.

## Abbreviations

CV: Contingent Valuation
DCE: Discrete Choice Experiment
MCDA: Multi-Criteria Decision Analysis
CJ: Citizens’ Jury
