# Supplementary material for: Investigating public values in health care priority – Chileans´ preference for national health care
Source: BMC Public Health. 2021 Feb 27;21:416. doi: 10.1186/s12889-021-10455-y (PMC7912507; doi:10.1186/s12889-021-10455-y)
Supplement: Supplementary file 1 — Additional file 1. Survey. [file 12889_2021_10455_MOESM1_ESM.docx]

**Appendix – Survey***

1. **Priority Setting**

Please decide what health program should have a relative higher priority in Chile from the following list of programs:

**Program A:** Investment in new health care facilities to provide easier access (closer distance to places where survey subjects live) and reduce travel time.

**Program B**: More generous insurance coverage (FONASA, ISAPREs, etc) such as for prescription drugs, lab exams, alternative medicine and medical attention.

**Program C:** Increase in the number of physicians and specialists available and improve their communication with patients.

**Program D**: Investment in information systems that make reservation for appointment easier and faster.

**Program E:** Improve the distribution of health care and public health awareness programs (eg. oral health, sexual and reproductive health, mental health, etc.) to all regions and better dissemination of those programs.

**Program F:** Improve availability of prescription drugs in all health care facilities and pharmacies.

**(SEE PROGRAMS CARD)**

Please prioritize each one of these 6 programs. You can assign more than one program in the same priority but each program can only be assigned once. Priority 1 means the most important program(s), then priority 2 means the secondary important program(s), and so forth. Stop once all programs are assigned.

**(SEE PRIORITY CARD)**

Now, we want to find more about the differences in your priorities. Assume that you have 18 points to allocate to these 6 programs to decide how to improve the healthcare services in Chile. Please decide how to allocate the 18 points or 18 stickers, remembering that this will have an impact on health care access. In the following question, the amounts you write down must add up to 18 points; e.g. you may decide to allocate 3 points to Program A (3 stickers); 15 points to Program B (15 stickers); and nothing to Program C, D, E and F (0 stickers).

To have an impact on health care services you should assign at least 3 points or 3 stickers. However, the more points you allocate to that program the better improvement we get (i.e. you can allocate more than 3 stickers to any program). Remember in order to improve a program the minimum requirement are 3 stickers (if you allocate more than 3 stickers in one program, that means there would be one program that gets no sticker).

**(SEE POINTS CARD)**

1. **Distributive Justice Principle for Health Care:**

There are four common principles for distributive justice in health care that many countries applied for priority setting in their health care system. They are: 1) equal access for health care, 2) equal access for equal health needs, 3) equal access for equal ability to benefit from health care and 4) equality in health. Access means people are being provided medical services independent of their income, age, sex, place of living, etc.

For example:

There are three persons A, B and C. There is one donated heart available for these three people, two of them need heart transplant.

Person A: A 35 yrs. old female who has heart failure that need a heart transplant to survive, she does not have any other health conditions.

Person B: A 75 yrs. old male who has heart failure that need a heart transplant to survive, in addition he also has diabetes, high blood pressure, high blood cholesterol and Alzheimer's disease.

Person C: A 20 yrs. old female who is healthy and does not need a heart transplant to survive.

Based on each of these 4 principles a society may come up with different solutions for who should get this heart.

1. **Equal access for health care**: Everyone irrespective whether they are healthy or sick should have equal access to health care.

*Solution*: Based on this principle persons A, B and C should have equal opportunity to receive this heart.

1. **Equal access for equal health needs**: Only people who are equally sick should have equal access to health care but not everyone.

*Solution*: Based on this principle, persons A and B should have equal opportunity to receive this heart, but not C.

1. **Equal access for equal ability to benefit from health care**: The higher priority to receive the medical service is for people who can have a healthier and longer life after they receive the medical services.

*Solution*: Based on this principle, person A will get the transplant (higher health benefits) because she is healthier and can live longer and healthier life after transplant than person B. Person C does not need the heart transplant (no benefits).

1. **Equality in health**: this is a complex principle, it depends on how we measure health. Let´s assume that we measure health in life-years, then this principle argues that everyone should live a similar number of life years (eg. 100 years). Based on this principle, health care services priority will be given to younger people so they can live also 100 years.

*Solution*: Based on this principle, the allocation is dependent on how we measure health. If we measure health in term of life years then person A should get the heart because she has lived only 35 years while person B already lived 75 years. Person C`s health will not be improved by the heart transplant. (However, if we change the measurement of health the solution may differ).

What do you think should be the distributive justice principle for the Chilean health care system that will guide the priority of the health care services?

**(SEE DISTRIBUTIVE JUSTICE CARD)**

1. **Opinion**

Should the Ministry of Health ask the Chilean people opinion about major health care system change? (Such as the kind of questions we ask you in this survey.)

Yes ____ No ____ Don’t know ___ Indifferent ___

* Cards have not been provided because they are not significant for this manuscript. The original survey is in Spanish.
