# Supplementary material for: Investigating public values in health care priority – Chileans´ preference for national health care
Source: BMC Public Health. 2021 Feb 27;21:416. doi: 10.1186/s12889-021-10455-y (PMC7912507; doi:10.1186/s12889-021-10455-y)
Supplement: Supplementary file 2 — Additional file 2. [file 12889_2021_10455_MOESM2_ESM.docx]

**Programs Card**

| A | **Program A.** Infrastructure | | B | **Program B.** Insurance coverage | |
| --- | --- | --- | --- | --- | --- |
|  | | 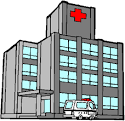 |  | | 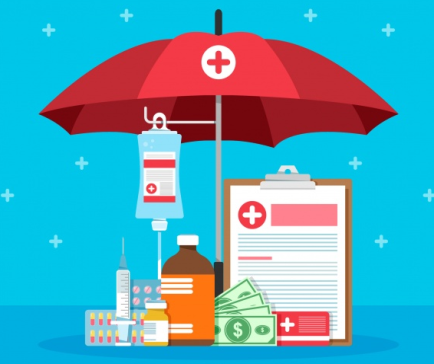 |
|  | | |  | | |
| C | **Program C.** Physicians and Specialists | | D | **Program D.** Information Systems | |
|  | | 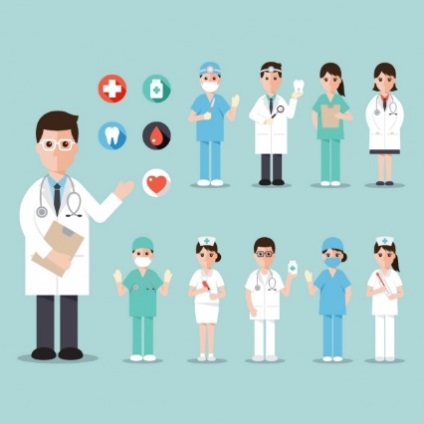 |  | |  |
|  | | |  | | |
| E | **Program E.** Health awareness programs | | F | **Program F.** Prescription drugs | |
|  | |  |  | | 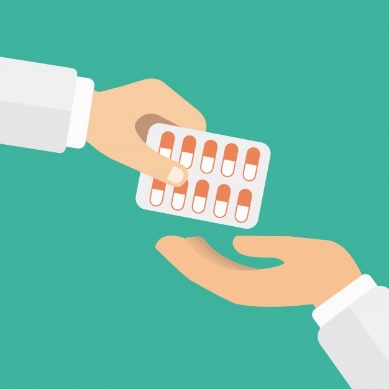 |
|  | | |  | | |

**Priority Card**

Please prioritize each one of these 6 programs. You can assign more than one program in the same priority but each program can only be assigned once. Priority 1 means the most important program(s), then priority 2 means the secondary important program(s), and so forth. Stop once all programs are assigned.

| **LEVEL**  **OF**  **IMPORTANCE** |  | | | |
| --- | --- | --- | --- | --- |
|  |  | **Priority** |  |  |
|  | Most important | **1** |  | assign 1-6 program(s) |
|  |  | **2** |  | assign 0-5 program(s),  do not repeat those assigned in Priority 1 |
|  |  | **3** |  | assign 0-4 program(s),  do not repeat those assigned in Priority 1 and 2 |
|  |  | **4** |  | assign 0-3 program(s),  do not repeat those assigned in Priority 1, 2 and 3 |
|  |  | **5** |  | assign 0-2 program(s),  do not repeat those assigned in Priority 1, 2, 3 and 4 |
|  | Less important | **6** |  | assign 0-1 program,  do not repeat those assigned in Priority 1, 2, 3, 4 and 5 |
|  |  | | | |

**Points Card**

Remember to improve a program the minimum requirement are 3 stickers (if you allocate more than 3 stickers in one program, that means there would be one program that gets no sticker).

| **No improvement** | **Minimal visible improvement** | **Major improvements** |
| --- | --- | --- |
| **Less than 3 stickers** | **3 stickers** | **More than 3 stickers** |

| **Program** |  | **Points** | | Total: |
| --- | --- | --- | --- | --- |
| **A** | **Infrastructure** |  |  |  |
| **B** | **Insurance coverage** |  |  |  |
| **C** | **Physicians and specialists** |  |  |  |
| **D** | **Information systems** |  |  |  |
| **E** | **Health awareness programs** |  |  |  |
| **F** | **Prescription drugs** |  |  |  |

**Stickers**

| **1** | **1** | **1** |
| --- | --- | --- |
| **1** | **1** | **1** |
| **1** | **1** | **1** |
| **1** | **1** | **1** |
| **1** | **1** | **1** |
| **1** | **1** | **1** |

**Distributive Justice Card**

What do you think should be the distributive justice principle for the Chilean health care system that will guide the priority of the health care services?

| **Option** | **Description** | **A** | **B** | **C** |  |
| --- | --- | --- | --- | --- | --- |
| **1** | **Equal access for health care** | 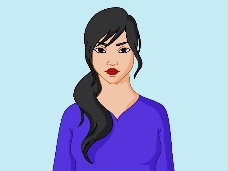 | 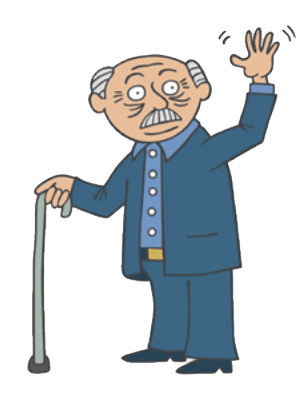 | 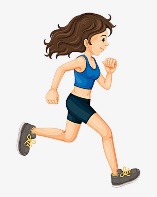 |  |
| **2** | **Equal access for equal health needs** | 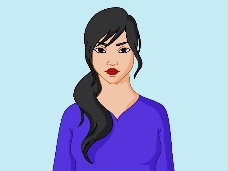 | 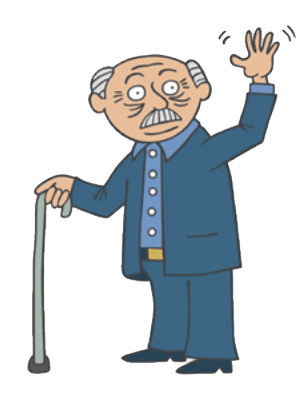 |  |  |
| **3** | **Equal access for equal ability to benefit from health care** | 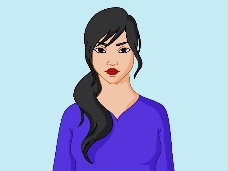 |  |  |  |
| **4** | **Equality in health** | 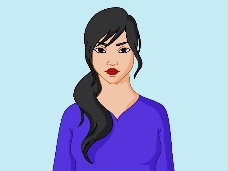(*) |  |  |  |
|  |  | **(*) (It depends on how you measure health)** | | |  |

| **Person A** | | |
| --- | --- | --- |
| 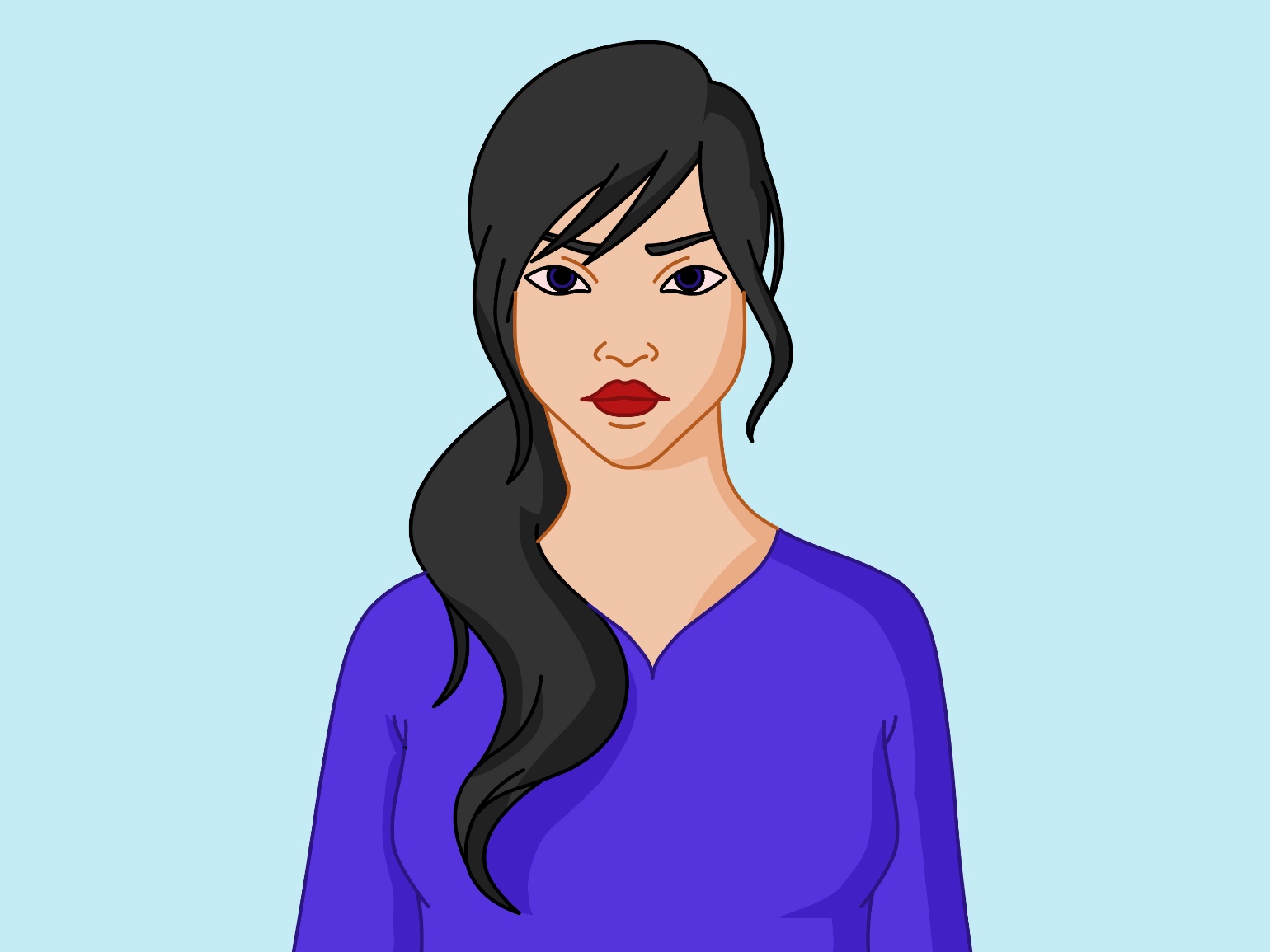 | | |
| **Age: 35 yrs. old, female**  **Health problem: Heart failure (need a heart transplant to survive)** | | |
| **Person B** | |  |
| 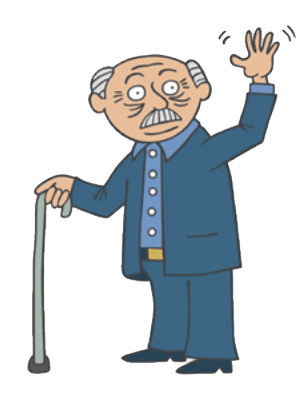 | |  |
| **Age: 75 yrs. old, Male**  **Health problems:**   - **Heart failure (need a heart transplant to survive)** - **Diabetes** - **High blood pressure** - **High blood cholesterol** - **Alzheimer´s disease** | |  |
| **Person C** |  |  |
| 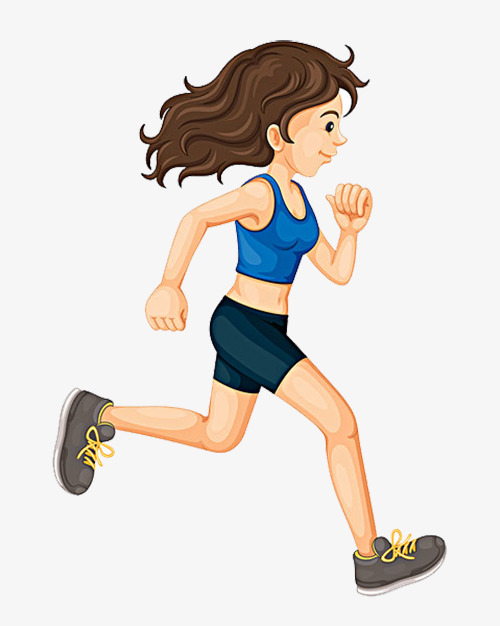 |  |  |
| **Age: 20 yrs. old, Female**  **Health problem: None (does not need a heart transplant to survive)** |  |  |
